# Comprehensive Proteomic Analysis of Lysine Acetylation in *Nicotiana benthamiana* After Sensing CWMV Infection

**DOI:** 10.3389/fmicb.2021.672559

**Published:** 2021-05-17

**Authors:** Bowen Yuan, Tingting Liu, Ye Cheng, Shiqi Gao, Linzhi Li, Linna Cai, Jian Yang, Jianping Chen, Kaili Zhong

**Affiliations:** ^1^State Key Laboratory for Managing Biotic and Chemical Threats to the Quality and Safety of Agro-products, Key Laboratory of Biotechnology in Plant Protection of Ministry of Agriculture and Zhejiang Province, Institute of Plant Virology, Ningbo University, Ningbo, China; ^2^Yantai Academy of Agricultural Science, Yantai, China

**Keywords:** CWMV, lysine acetylation, chloroplast, photosynthesis, histone 3

## Abstract

Protein lysine acetylation (Kac) is an important post-translational modification mechanism in eukaryotes that is involved in cellular regulation. To investigate the role of Kac in virus-infected plants, we characterized the lysine acetylome of *Nicotiana benthamiana* plants with or without a Chinese wheat mosaic virus (CWMV) infection. We identified 4,803 acetylated lysine sites on 1,964 proteins. A comparison of the acetylation levels of the CWMV-infected group with those of the uninfected group revealed that 747 sites were upregulated on 422 proteins, including chloroplast localization proteins and histone H3, and 150 sites were downregulated on 102 proteins. Nineteen conserved motifs were extracted and 51 percent of the acetylated proteins located on chloroplast. Nineteen Kac sites were located on histone proteins, including 10 Kac sites on histone 3. Bioinformatics analysis results indicated that lysine acetylation occurs on a large number of proteins involved in biological processes, especially photosynthesis. Furthermore, we found that the acetylation level of chloroplast proteins, histone 3 and some metabolic pathway-related proteins were significantly higher in CWMV-infected plants than in uninfected plants. In summary, our results reveal the regulatory roles of Kac in response to CWMV infection.

## Introduction

Protein post-translational modifications (PTMs) are dynamic and reversible protein processing events that occur in prokaryotic and eukaryotic cells (Rao et al., 2014). Previous studies shown that PTMs involved in plenty of biological processes, including gene expression, enzymatic activity, protein stability and cell signaling (Khoury et al., 2011). To date, more than 450 distinct PTMs have been identified, including acetylation, succinylation, malonylation, butyrylation, and propionylation. Lysine residues acetylation, is an important regulatory mechanisms that broaden and fine-tune protein functions (Xing and Poirier, 2012). Furthermore, protein acetylation plays an important role in cellular biological activities, such as cell growth, apoptosis, cytokinetics, cell metabolism (Choudhary et al., 2009; Wang et al., 2010), enzymatic activity (Starai and Escalante-Semerena, 2004; Nambi et al., 2013), protein interactions (Choudhary et al., 2009), protein stability (Liang et al., 2011), and metabolic pathways. The balance of lysine acetylation depends on the regulation of specific lysine acetyl-acetylases and deacetylases (Choudhary et al., 2009; Xing and Poirier, 2012; Konig et al., 2014; Hartl et al., 2017).

Protein lysine acetylation (Kac) of histones was first confirmed to control gene expression and regulate epigenetic in 1963 (Phillips, 1963). Early studies of protein acetylation in *Arabidopsis* and rice (*Oryza sativa*) reported that multi-lysine residues are acetylated in histone H3, including K4, K9, K14, K18, K23, K27, and K36. Lysine residues in histone H4, such as K5, K8, K12, K16, and K20 are acetylated (Benhamed et al., 2006; Fuchs et al., 2006; Hollender and Liu, 2008; Servet et al., 2010; Tan et al., 2011; Xue et al., 2018). Recently, with the development of proteomic techniques more and more modifications have been discovered, including non-histone acetylation. For example, a lot of Kac proteins in rice was found, including 1,669 Kac sites on 1,024 proteins in vegetative cells and reproductive organs (Li et al., 2018) and 1,337 Kac sites on 716 proteins in whole seedlings (Xiong et al., 2016). In addition, 2,057 Kac sites on 1,022 proteins have been identified in *Arabidopsis* (Hartl et al., 2017), including many respiratory chain proteins and tricarboxylic acid cycle enzymes. In addition to acetylated proteins mentioned above, many have other functions, such as photorespiration, regulating redox and metabolism (Konig et al., 2014). Kac proteins have also been identified in strawberry (*Fragaria × ananassa*) (Fang et al., 2015), grape (*Vitis vinifera*) (Melo-Braga et al., 2012), and wheat (*Triticum aestivum*) (Zhang et al., 2016; Table 1). However, compared with rice and *Arabidopsis*, acetylomes of *Nicotiana benthamiana* and of chinese wheat mosaic virus (CWMV)-infected *N. benthamiana* plants have been poorly studied. Although there are several studies about lysine acetylation in plant, the impact and regulation on pathogen interactions, particularly virus infection, has not been investigated in plants to date (Hartl et al., 2017).

**TABLE 1:** Lysine acetylomes identified in plants.

**Species**	**Number of identified proteins**	**Number of identified sites**	**References**
*Arabidopsis*	57	64	Wu et al., 2011
*Arabidopsis*	74	91	Finkemeier et al., 2011
*Arabidopsis*	204	348	Konig et al., 2014
*Arabidopsis*	1,022	2,057	Hartl et al., 2017
*Oryza sativa*	44	60	Nallamilli et al., 2014
*Oryza sativa*	389	699	He et al., 2016
*Oryza sativa*	716	1,337	Xiong et al., 2016
*Oryza sativa*	1,024	1,669	Li et al., 2018
*Oryza sativa*	866	1,353	Xue et al., 2018
*Strawberry Stigmata*	684	1,392	Fang et al., 2015
*Vitis vinifera*	97	138	Melo-Braga et al., 2012
*Triticum aestivum*	277	416	Zhang et al., 2016

CWMV belong to family *Virgaviridae*, the genus *Furovirus* (Adams et al., 2009), and causes a damaging disease on wheat with light chlorotic streaking of young leaves. In old leaves, CWMV infection cause bright-yellow chlorotic streaking or even purple chlorotic stripes are observed. Severely infected plants become stunted, wilt, and eventually die (Chen, 1993; Diao et al., 1999). CWMV Has a bipartite positive-sense single-stranded RNA genome and its particle is rigid rod-shaped (Diao et al., 1999). There are three proteins encoded by RNA1 (7,147 nt), including a 153-kDa replicase protein, a 212-kDa protein contain a RNA-dependent RNA polymerase domain, and cell-to-cell movement protein. There are four proteins encoded by RNA2 (3,564 nt), including a major coat protein, two minor CP-related proteins, and a 19 kDa cysteine-rich RNA silencing suppressor (Diao et al., 1999; Andika et al., 2013). Recently, the CWMV infectious cDNA clones have been developed (Yang et al., 2016), making investigations of CWMV and the CWMV-host interaction possible. However, to date, only a few host factors have been identified to interact with CWMV encoding proteins and involved in the CWMV infection, and no PTM of the host protein related to the CWMV infection response has been identified.

In this study, we explored lysine acetylation in CWMV-infected *N. benthamiana* leaves. We used 2D-LC-MS/MS and advanced bioinformatic analyses to enrichment Kac peptides, and to determine the Kac landscape in *N. benthamiana*. In total, our analysis yielded 4,803 acetylated lysine sites on 1,964 proteins. Further proteomic analysis revealed that the acetylated proteins involved in varied biological processes, cellular functions and subcellular localization. Among these, most upregulated Kac proteins are involved in photosynthetic processes whereas the downregulated Kac proteins participate in metabolic processes. This study have broaden the understanding of acetylomes in *N. benthamiana* and reveals a lot of acetylation sites involved in specific defense processes against virus infection.

## Experimental Procedures

### CWMV Inoculation and Plant Growth

For the agroinfiltration of *Nicotiana benthamiana* leaves, we use electroporation method to transform the plasmids into *Agrobacterium tumefaciens* strain (GV3101). Individual bacterial cultures with plasmid were grown overnight, collected, and resuspended in the induction buffer (100 mM acetosyringone, 1 M MgCl_2_, 10 mM MES, pH 5.6). The bacterial cultures were incubation for 3 h at room temperature, and then infiltrated individually into two leaves of four-leaf-stage *N. benthamiana* using needleless syringes. Three plants were infiltrated with the bacterial culture. Three plants inoculated with induction buffer only were used as control plants. After inoculation, *N. benthamiana* plants were grown at 15∘C and under 16/8 h light/dark conditions for 14 days.

### *Nicotiana benthamiana* Protein Extraction

Sample proteins were extracted from 1 g leaves. Firstly, we ground samples to form powder in liquid nitrogen and then add lysis buffer (1% protease inhibitor cocktail, 8 M urea, 1% Triton-100, 10 mM dithiothreitol, 3 μM trichostatin A and 50 mM nicotinamide for acetylation) to the powdered sample, which was then placed on ice and sonicated using ultrasonic crusher (Scientz, Ningbo, China) for three times. Secondly, the suspension was centrifuged at 20,000 *g* for 10 min at 4∘C to remove the remaining debris. Thirdly, the protein was precipitated by incubating the lysate with cold 20% trichloroacetic acid for 2 h at –20∘C. The precipitate was collected after 12,000 *g* centrifugation for 10 min at 4∘C and then washed with cold acetone for three times. Finally, the protein was dissolved in 8 M urea and the protein concentration was determined using a BCA kit according to the manufacturer’s instructions.

### Database Search

The resulting 2D-LC-MS/MS data were processed using the MaxQuant search engine (v. 1.5.2.8). Tandem mass spectra were searched against the *Nicotiana tabacum* database^1^ concatenated with a reverse decoy database. We set the mass tolerance as 20 ppm in “First search,” the “Main search” set as 5 ppm, and set fragment ions as 0.02 Da. The false discovery rate was adjusted to lower than 1% and the minimum score for modified peptides was set as > 40.

### Bioinformatics Analysis

For bioinformatics analysis, we use the UniProt-GOA database^2^ to unify the gene and gene product attributes and to assimilate and disseminate annotation data of acetylation-modified peptides and proteins. Next, we use Gene Ontology (GO) annotation to classify identified proteins into three categories: molecular function, biological process and cellular component. The corrected *p* < 0.05 was considered to be significant. The online KEGG database^3^ was used for pathway and protein domain analyses of the identified *N. benthamiana* proteins. The corrected *p* < 0.05 was considered to be significant in pathway. These pathways were classified into hierarchical categories according to the KEGG website.

InterProScan software was used to annotate the functional description of the identified protein domains based on the InterPro^4^ domain database and the protein sequence alignment method. The InterPro database and two-tailed Fisher’s exact test was used for each protein category. The corrected *p* < 0.05 of protein domains was considered significant. The subcellular localization of all peptides and identified proteins was predicted in Wolfpsort software.

The Kac amino acids positions motif of modifier-21-mers in all protein sequences was analyzed and displayed using motif-x software. All the database protein sequences were used as background database parameters; default values were used for other parameters.

### Protein–Protein Interaction Network

The protein-protein interaction analysis is based on STRING database (version 11.0) and only select the searched proteins that present interactions in the dataset. Set a confidence score > 0.7 between all interactions to define the interaction confidence. The densely connected regions of searched proteins was analyzed by a graph theoretical clustering algorithm and plug-in tool kit part molecular complex detection (MCODE).

### Protein Expression

To investigate the acetylation of proteins A0A1S4CHI1, A0A1S4BE66, and A0A1S4CLZ9, these proteins were fused with a green fluorescent protein (GFP) tag at the C-terminus. The proteins were expressed in CWMV-infected and uninfected *N. benthamiana* leaves. Forty eight hours after inoculation, green fluorescence was observed under a confocal fluorescence microscope (Nikon, Tokyo, Japan; A1 + A1R) before extracting proteins from leaves.

### Western Blot Analysis

Total proteins were extracted from 0.1 g fresh leaf tissues by ground them in liquid nitrogen and then add protein extraction buffer (1% Triton-100, 10 mM dithiothreitol and 1% protease inhibitor cocktail). The mixture liquid was centrifuged at 18,000 *g* for 10 min at 4∘C. Next, collect 160 μl supernatant of each sample and add 40μl SDS-PAGE buffer, then boiled in at 100∘C for 10 min. The supernatant was loaded onto a 4–20% SDS-PAGE gel, followed by western blot analysis using Histone H3ac (pan-acetyl) antibody (rabbit, Active Motif, Carlsbad, CA, United States), anti-acetyl-lysine antibody (mouse, PTM Biolabs, Hangzhou, China), histone H3K9ac (pan-acetyl) antibody (rabbit, Active Motif), histone H3K18ac (pan-acetyl) antibody (rabbit, Active Motif), histone H3K27ac (pan-acetyl) antibody (rabbit, Active Motif), histone H3K79ac (pan-acetyl) antibody (rabbit, Active Motif).

## Results

### Proteome-Wide Analysis of Lysine Acetylation Peptides and Proteins in CWMV-Infected *N. benthamiana*

**FIGURE 1:**
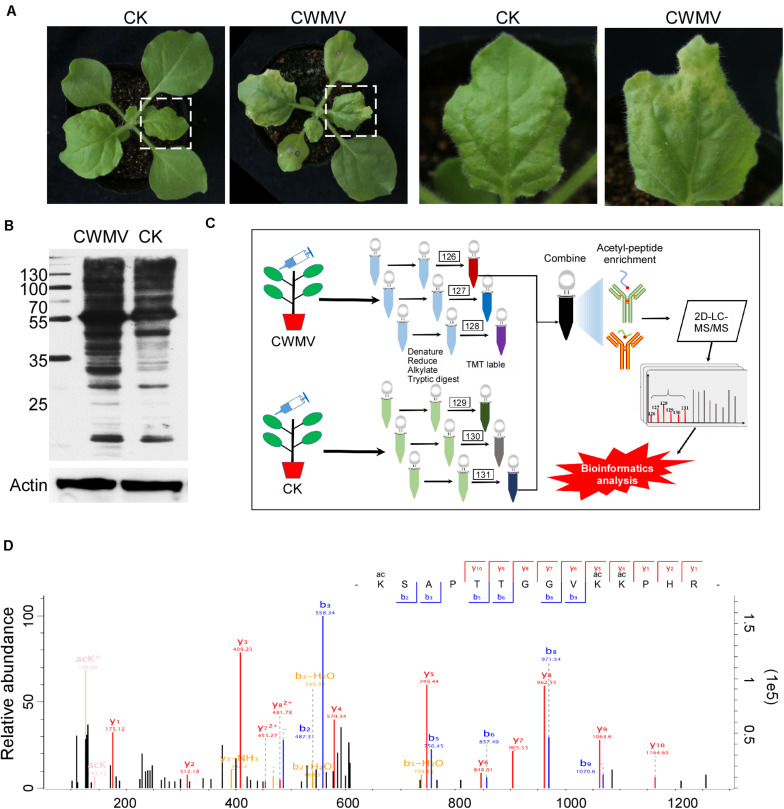
Proteome-wide analysis of lysine acetylation peptides and proteins. **(A)** Photograph of *Nicotiana benthamiana* 14 days post-inoculation with CWMV. **(B)** Detection of Lys-acetylated proteins in CWMV-infected *N. benthamiana* leaf samples and uninfected leaf samples by western blot assays using anti-acetyl-Lys antibodies. **(C)** The workflow used for the Kac proteomic experiments. **(D)** LC-MS/MS spectra of acetyl-peptides from a upregulated chloroplastic protein named 29 kDa ribonucleoprotein B; acetyl-peptide VEVIYDK(ac)LTGR with one acetylation site at K122.

**FIGURE 2:**
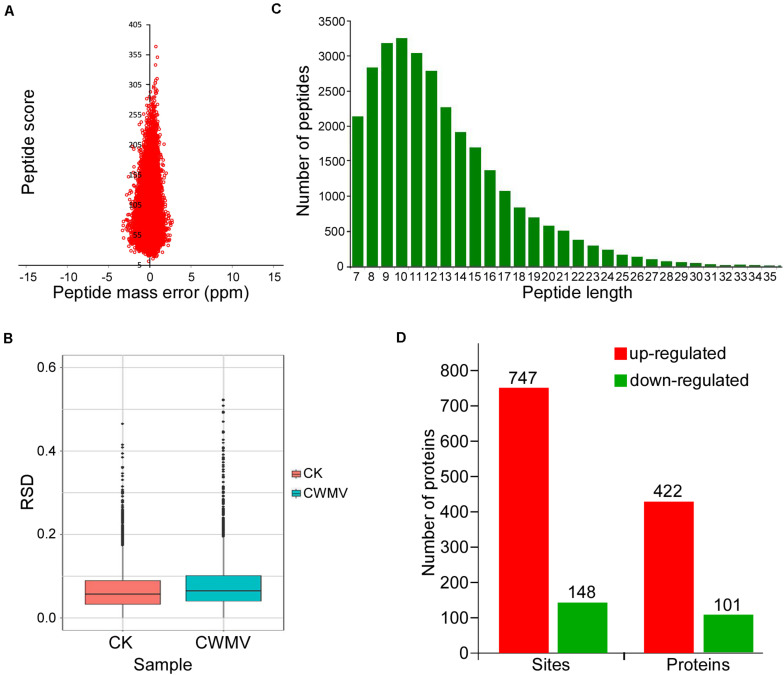
Distribution and motif analysis of lysine acetylation sites. **(A)** Mass error distribution of all identified peptides. **(B)** Boxplot showing the sequence coverage of proteins that were quantified under each extraction condition. RSD, relative standard deviation coefficient. **(C)** Distribution of peptide length. **(D)** Number of lysine acetylation sites on peptides and proteins identified in CWMV-infected *N. benthamiana* leaf samples and uninfected leaf samples.

*Nicotiana benthamiana* leaves infected with CWMV show some physiological changes (Figure 1A and Supplementary Figure 1). To determine the overall number of acetylated proteins and to compare protein acetylation levels in CWMV*-*infected *N. benthamiana* leaves 14 days post infection with those of uninfected leaves, a western blot assay was performed using protein extracts from *N. benthamiana* leaves with or without CWMV infection. Multiple lysine-acetylated protein bands were detected using anti-acetyl-lysine antibodies; and then, the CWMV-infected leaves showed stronger reactions to the anti-acetyl-lysine than the uninfected control leaves (Figure 1B). To analyze the lysine acetylation peptides, we performed LC-MS/MS analysis (Figure 1C). LC-MS/MS spectra of acetylated peptides (upregulation of chloroplastic protein 29 kDa ribonucleoprotein B) are shown as an example (Figure 1D). To confirm the validity of the LC-MS/MS data, we checked the quality errors of acetyl-peptides and sample repeatability. The distribution of quality errors and the relative standard deviation coefficient (RSD) were close to zero (Figures 2A,B), which confirmed that the LC-MS/MS data were highly accurate. The length of most acetylation peptides ranged from 7 to 25 (Figure 2C). In total, our analysis yielded 4,803 acetylated lysine sites on 1,964 *N. benthamiana* proteins. A comparison of the acetylation levels of the CWMV-infected group with those of the uninfected group revealed that 747 sites were upregulated on 422 proteins and 150 sites were downregulated on 102 proteins when using a change threshold of at least 1.2 times and a *t*-test *p* < 0.05 (Figure 2D). Among all the acetylation level changed proteins, 19 Kac sites were combined in core histones, including 10 Kac sites on histone H3, and 278 Kac sites were combined in chloroplast proteins (Supplementary Table 1).

### Distribution Characterization and Motif Analysis of Acetylation Sites

**FIGURE 3:**
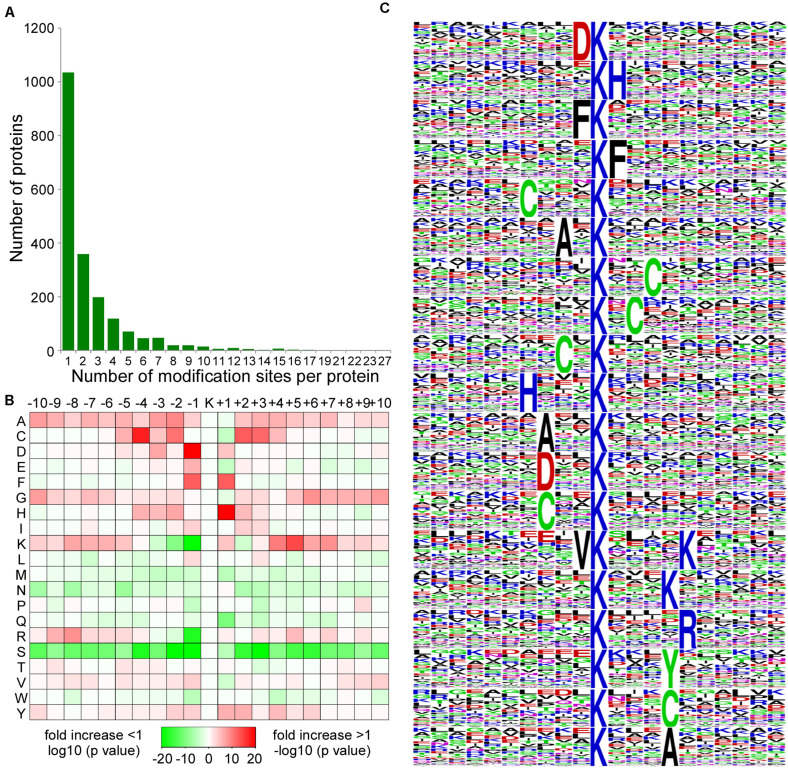
Distribution and enrichment analysis of lysine acetylation sites. **(A)** The number of acetylation sites on acetylated proteins. **(B)** Enrichments of amino acids around the acetylation sites. The intensity map shows the relative abundance for 10 amino acids from the lysine-acetylated site. The colors in the intensity map represent the log10 of the ratio of frequencies within acetyl-21-mers vs. non-acetylated (red shows enrichment, green shows depletion). **(C)** Sequence probability logos of significantly enriched acetylation site motifs for ± 10 amino acids around the lysine acetylation sites. The size of each letter corresponds to the frequency of that amino acid residue in that position.

To evaluate the number of acetylation sites per protein in CWMV-infected *N. benthamiana* leaves, we calculated the acetylation sites on proteins. The results indicated that 52.7% (1,035/1,964) contained a single acetylation site, 18.3% (359/1,964) contained two acetylation sites, 10.1% (198/1,964) contained three acetylation sites, and 18.9% (372/1,964) contained more than three acetylation sites (Figure 3A). Furthermore, 53 proteins contained at least 10 acetylation sites, including Q76N23, histone H3, which contained 10 acetylation sites, A0A1S4CB32, a chloroplastic heat shock-related protein, which contained 22 acetylation sites, and A0A1S3Y0T4, an elongation factor 2, which contained 27 acetylation sites (Supplementary Table 2).

To further evaluate the characteristics and particular domain of acetylation sites, we analyzed the motif of sequences comprising lysine residues and amino acids in specific positions by using Motif-x program. A position-specific intensity map was generated to assess the significantly enriched amino acids around the acetylation sites. The concentration of motif with amino acid frequencies showed that the + 1, + 2, or + 3 positions have a preference for H, F, or C residues and that the –1, –2, or –3 positions have a preference for A, C, D, F, or V residues. However, serine residues were significantly lacking from all 20 positions around acetylation sites (Figure 3B). A total of 19 conserved motifs in D^∗^Kac, Kac^∗^H, H^∗^Kac, F^∗^Kac, Kac^∗^F, C^∗^Kac, Kac^∗^C, A^∗^Kac, Kac^∗^A, V^∗^Kac^∗^K, Kac^∗^R, Kac^∗^K, and Kac^∗^Y were summarized from 3,360 acetylated peptides. Among these motifs, D^∗^Kac represented 379 (11.3%) of the enrichment motifs to be the most common combination (Figure 3C). These newly identified amino acid residues surrounding acetylation sites in *N. benthamiana* could potentially provide acetylation binding sites for future studies.

### Functional Distribution and Subcellular Localization of Acetylation Proteins in CWMV-Infected *N. benthamiana*

**FIGURE 4:**
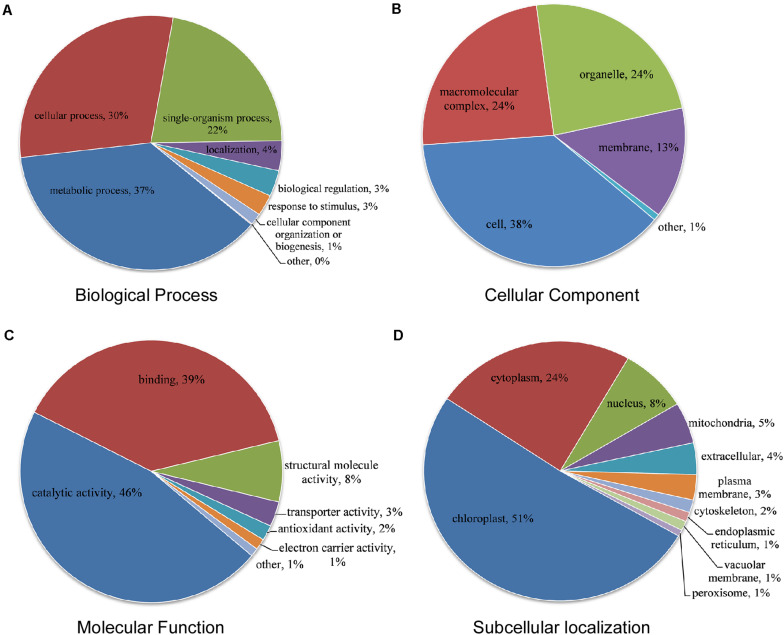
Functional distribution and subcellular localization of lysine acetylated proteins in CWMV-infected *N. benthamiana*. **(A)** Lysine acetylated proteins classification according to their biological process. **(B)** Lysine acetylated proteins classification based on their cellular component. **(C)** Lysine acetylated proteins classification according to their molecular function. **(D)** Subcellular localization of lysine acetylated proteins.

We detected 524 novel acetylated proteins in CWMV-infected *N. benthamiana*. To better understand the potential functions of these proteins, we further assessed the GO analysis using DAVID (Huang da et al., 2009). Proteins were categorized based on their biological process, cellular component, and molecular function. We found that 37% of acetylated proteins were involved in metabolic process, 30% in cellular process, and 22% in single-organism process according to biological process category (Figure 4A). There are 38% of acetylated proteins were located in the cell, 24% in a macromolecular complex, 24% in organelles, and 13% in the membrane according to the cellular component category (Figure 4B). In the molecular function category, most acetylated proteins were either associated with catalytic activity (46%) or binding (39%) (Figure 4C). These results indicated that the enzyme metabolism related proteins might be a high probability of acetylation in CWMV-infected *N. benthamiana*. For the subcellular localization, we found that most acetylation proteins were located in the chloroplast (51%) in CWMV-infected *N. benthamiana* (Figure 4D). In addition, some of the remaining acetylation proteins were mainly located in the cytoplasm (24%), nucleus (8%), and mitochondria (5%), respectively.

### Enrichment Analysis and Domain Enrichment of Lysine-Acetylated Proteins in CWMV-Infected *N. benthamiana* Plants

**FIGURE 5:**
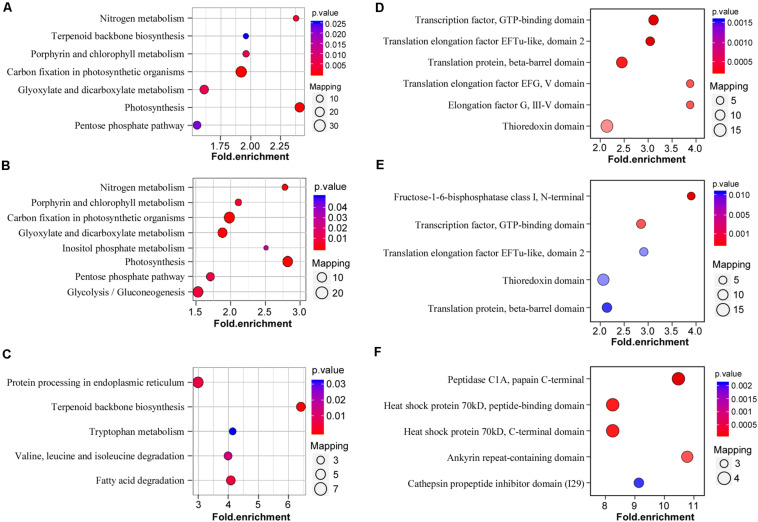
Enrichment analysis and domain enrichment of lysine-acetylated proteins in CWMV-infected *N. benthamiana* plants. **(A)** KEGG pathway-based enrichment analysis of proteins in CWMV-infected versus uninfected (CK) plants (–log_10_ *p* > 1.5). **(B)** KEGG pathway enrichment analysis of upregulated proteins in CWMV-infected versus CK plants (–log_10_ *p* > 1.5). **(C)** KEGG pathway-based enrichment analysis of downregulated proteins in CWMV-infected versus CK plants (–log_10_ *p* > 1.5). **(D)** Protein domain enrichment analysis of CWMV-infected versus CK plants. **(E)** Protein domain enrichment analysis of upregulated proteins in CWMV-infected versus CK plants. **(F)** Protein domain enrichment analysis of downregulated proteins in CWMV-infected versus CK plants.

To investigate the general functions of acetylation proteins in the *N. benthamiana* response to CWMV infection, acetylated proteins detected in CWMV-infected versus uninfected (CK) plants were mapped to KEGG metabolic pathways. KEGG metabolic pathway analysis showed that most acetylated proteins participated in the regulation of photosynthesis, carbon fixation in photosynthetic organisms, nitrogen metabolism, porphyrin and chlorophyll metabolism, and glyoxylate and dicarboxylate metabolism (Figure 5A). The acetylation proteins participated in the regulation of photosynthesis, carbon fixation in photosynthetic organisms, glyoxylate and dicarboxylate metabolism, and nitrogen metabolism were upregulated (Figure 5B). Downregulated acetylated proteins participated in the regulation of terpenoid backbone biosynthesis, fatty acid degradation, and protein processing in the endoplasmic reticulum (Figure 5C).

To verify the lysine acetylation preferred targets, protein domains were analyzed using the InterPro domain database. The protein domain enrichment results revealed that 21 domains were enriched (Supplementary Table 3), mainly domains that included an elongation factor or translation elongation factor, and metabolic protein domains, such as the transcription factor GTP-binding domain, translation elongation factor EFTu-like domain 2, and translation protein beta-barrel domain (Figure 5D). Among these, approximately 20% of proteins were identified as chloroplast-related protein domains (Supplementary Table 4), suggesting that Kac regulate chloroplastic functions in response to CWMV infection. Meanwhile, fructose-1-6-bisphosphatase class I domain, transcription factor, GTP-binding domain and translation elongation factor EFTu-like domain 2 were enriched in upregulated lysine acetylated proteins (Figure 5E). Furthermore, the peptidase C1A papain C-terminal domain, heat shock protein peptide-binding domain, and heat shock protein 70 kDa, C-terminal domain were enriched in downregulated lysine acetylated proteins (Figure 5F).

### Analysis of the Interaction Network of Acetylated Proteins

**FIGURE 6:**
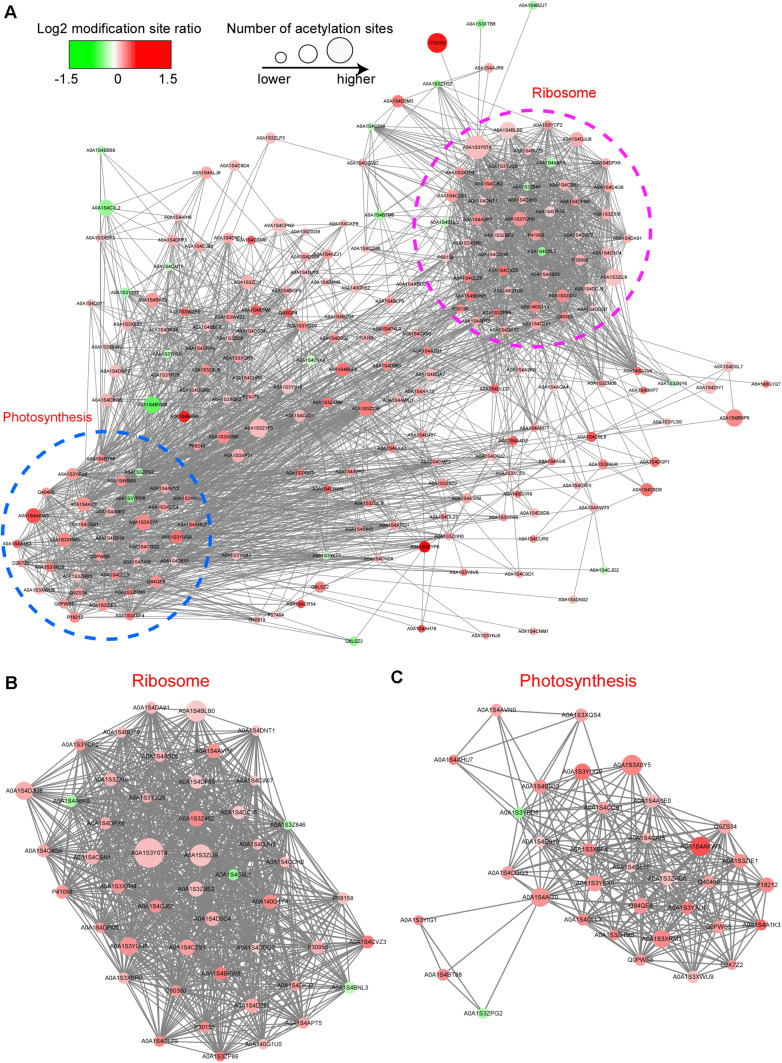
Interaction networks of differentially expressed acetylated proteins in CWMV-infected plants compared with expression in uninfected *N. benthamiana* plants. **(A)** The entire protein–protein interaction network of acetylated proteins of CWMV-infected versus uninfected CK plants. **(B)** Interaction network of acetylated proteins associated with the ribosome of CWMV-infected versus uninfected CK plants. **(C)** Interaction network of acetylated proteins involved in the photosynthesis of CWMV-infected versus uninfected CK plants.

To further clarify whether the acetylated proteins are associated with the protein component and the crosslink between we used the STRING database^5^ to construct protein–protein interaction (PPI) networks for acetylated proteins. According to the STRING construct results, we used MCODE tool to obtain entire interaction network and extracted three highly interactive clusters from them. A comparison of CWMV-infected leaves with uninfected leaves revealed 200 acetylated proteins involved in interactions that mapped to the protein interaction database (Figure 6A and Supplementary Table 5). Of these, 178 were upregulated, including HSP70, psbO, PSBP2, RPL16 and RPL2, and 22 were downregulated. The top group consisted of acetylated ribosome proteins (Figure 6B). Furthermore, a comparison of CWMV-infected leaves with uninfected leaves revealed 34 acetylated photosynthesis-related proteins in interactions that mapped to the protein interaction database. These photosynthesis-related proteins could be classified into several groups, such as photosystem I related proteins, chlorophyll a/b binding proteins, photosystem II related proteins and ATP synthase proteins (Figure 6C and Supplementary Table 6).

### Acetylated Proteins Involved in Photosynthesis

**FIGURE 7:**
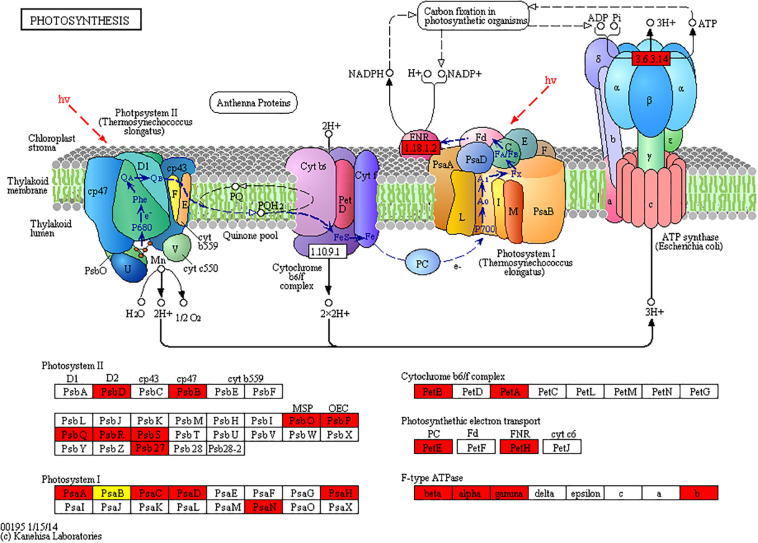
KEGG pathways of significantly enriched photosynthesis. The red highlights mean the acetylated proteins that were significantly upregulated in the photosynthesis pathway of CWMV-infected *N. benthamiana* plants compared with those of uninfected *N. benthamiana* (CK) plants. The yellow highlight means that acetylated protein with one lysine site downregulated and one sites upregulated in CWMV-infected plants.

Given that a large number of acetylated proteins associated with photosynthesis pathway were found by KEGG pathway enrichment analysis. Further analysis showed that photosystem II (PSII) protein complex members, including psbB, psbD, psbO, psbP, psbQ, psbR, and psbS, were identified as acetylated proteins in *N. benthamiana* and were upregulated in CWMV-infected plants. In addition, photosystem I (PSI) subunits, including PsaA, PsaB, PsaC, PsaD, PsaN, and PsaH, were also found to be acetylated. Among these, only one site on the PsaB subunit was downregulated, whereas the other sites were upregulated in CWMV-infected plants. Similarly, the acetylation level of some chloroplast ATP synthases, such as alpha, beta, gamma, and ATPF0B, were found to be upregulated in CWMV-infected plants (Figure 7 and Supplementary Table 7). These results indicate that CWMV infection induces acetylation of proteins associated with photosynthesis.

### Histone 3 and Chloroplastic Protein Acetylation Levels Were Upregulated in *N. benthamiana* After CWMV Infection

**FIGURE 8:**
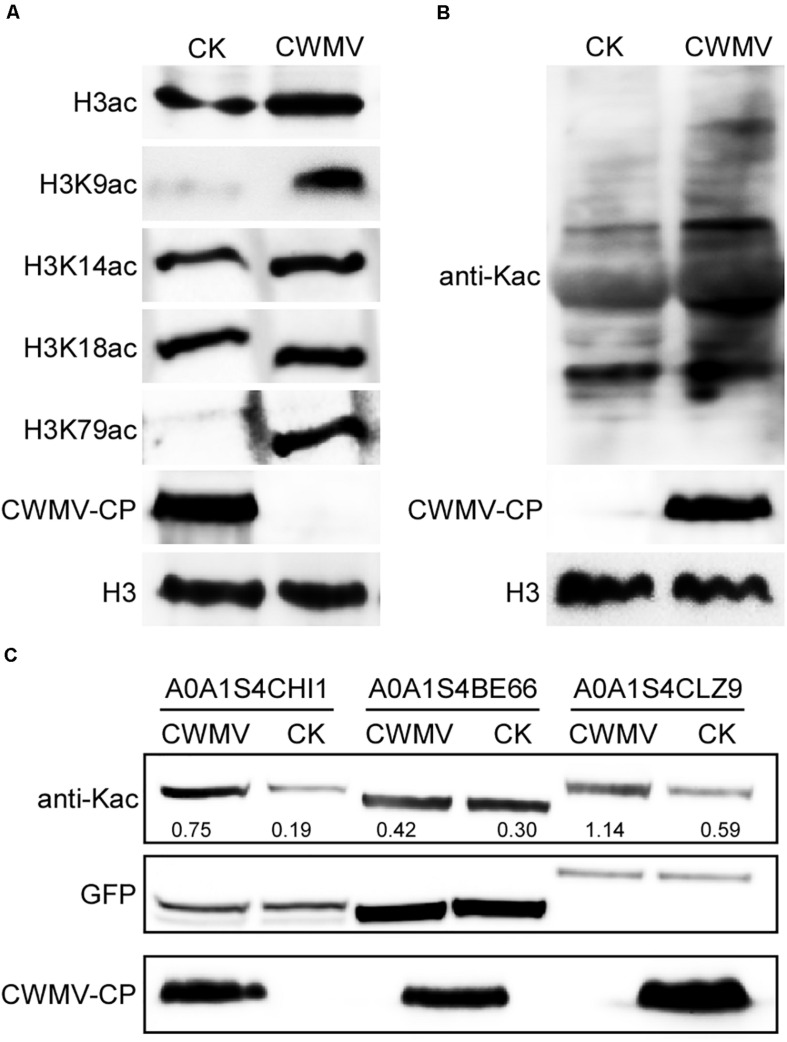
Protein acetylation levels in CWMV-infected and uninfected *N. benthamiana*. **(A)** Western blot analyses of different acetylated lysine sites on histone H3 in CWMV-infected and uninfected *N. benthamiana* leaf samples using H3K9ac-, H3K14ac-, H3K18ac-, and H3K79ac-specific antibodies. The expression level of CWMV-CP in the same samples was used to confirm the infection. The expression level of histone H3 in the same samples was used as an internal control. **(B)** Western blot analyses of chloroplast proteins extracted from CWMV-infected and the uninfected *N. benthamiana* leaves using anti-Kac antibodies. The expression level of CWMV-CP in the same samples was used to confirm the infection. The expression level of histone H3 in the same samples was used as an internal control. **(C)** Western blot analyses of selected proteins extracted from CWMV-infected and the uninfected *N. benthamiana* leaves using anti-Kac antibodies and anti-GFP antibodies. The expression level of CWMV-CP in the same samples was used to confirm the infection. The expression level of protein fused with GFP in the samples was used as an internal control.

To confirm that the acetylation level of histone 3 was upregulated in CWMV-infected *N. benthamiana* leaves compared with levels in uninfected leaves, a western blot assay was performed using protein extracts from *N. benthamiana* leaves with or without CWMV infection. Although an acetylated histone 3 protein band was detected for both CWMV-infected and uninfected *N. benthamiana* leaves, CWMV-infected leaves showed stronger reactions to anti-histone H3ac than uninfected control leaves. To confirm the candidate acetylation sites on H3, western blot assays were performed using anti-H3K9ac, anti-H3K14ac, anti-H3K18ac, and anti-H3K79ac antibodies. The analysis revealed that levels of H3K9ac and H3K79ac in CWMV-infected *N. benthamiana* leaves were obviously higher than those in uninfected *N. benthamiana* leaves. However, the levels of H3K14ac and H3K18ac were similar in CWMV-infected and uninfected *N. benthamiana* leaves (Figure 8A). In addition, we measured the acetylation level of chloroplast proteins by performing a western blot assay using chloroplast proteins extracted from *N. benthamiana* leaves with or without CWMV infection. Although multiple lysine-acetylated chloroplast protein bands were detected, CWMV-infected leaves showed stronger reactions to the anti-acetyl-lysine than uninfected control leaves (Figure 8B). Furthermore, we selected three upregulated lysine-acetylated proteins (A0A1S4CHI1, A0A1S4BE66, and A0A1S4CLZ9) at random from proteins identified as significantly upregulated when using a change threshold of at least 1.2 times and a *t*-test *p* < 0.05 (Supplementary Table 1). We measured the acetylation level of these proteins by performing a western blot assay using purified transient expression proteins extracted from *N. benthamiana* leaves with or without CWMV infection. A specific lysine-acetylated protein band was detected for all leaves. Although levels of A0A1S4CHI1 and A0A1S4CLZ9 lysine-acetylation were obviously higher in CWMV-infected *N. benthamiana* leaves than in uninfected *N. benthamiana* leaves, levels of lysine-acetylation in A0A1S4BE66 in CWMV-infected *N. benthamiana* leaves were only slightly higher than those in uninfected *N. benthamiana* leaves (Figure 8C). These results were confirmed by LC-MS/MS analyses (Supplementary Table 8). Our findings indicate that CWMV infection induced the acetylation of quite a few proteins in the host plant.

## Discussion

Lysine acetylation of proteins, a dynamic and reversible post-translational modification, plays a critical regulatory role in both eukaryotes and prokaryotes (Zhang et al., 2016). This is the first attempt to catalog acetylated proteins in *N. benthamiana* and the first insight into acetylation level changes in response to CWMV infection. Here, we detected 4,803 acetylated lysine sites on 1,964 proteins. These modified proteins in *N. benthamiana* are involved in different metabolic processes and participate in various biological processes and single-organism processes. Furthermore, the analysis of protein interaction network demonstrated that a wide range of interactions were modulated by lysine acetylation. These suggesting that protein acetylation might be a frequently event in plants associated with pathogen infection, responses to environmental stress, and metabolic processes. These results were similar to previous studies of acetylated proteins in plants, including rice (Nallamilli et al., 2014; He et al., 2016; Xiong et al., 2016; Li et al., 2018), strawberry (Fang et al., 2015), and *Arabidopsis* (Finkemeier et al., 2011; Wu et al., 2011; Konig et al., 2014; Hartl et al., 2017).

The chloroplast organelle in mesophyll cells of higher plants represents a sunlight-driven metabolic factory that eventually fuels life on our planet (Kirchhoff, 2019). In this study, a large proportion of acetylated proteins were located in chloroplasts (51%), suggesting that there is a close relationship between virus infection and chloroplast proteins acetylation. According to previous studies, chloroplasts could effectively activate the defensive hormonal responses in the process of plant-pathogen interaction and viral proteins located to chloroplast could promote viral pathogenesis. For example, the tomato yellow leaf curl virus-encoded C4 protein re-localizes from the plasma membrane to chloroplasts when the plant defense response is activated, interfering with chloroplast-dependent anti-viral salicylic acid biosynthesis (Medina-Puche et al., 2020). Previous studies have shown that the chloroplast provides a compartment for plant virus replication as well as membrane contents, and might help viruses evading the RNA-mediated defense response (Ahlquist et al., 2003; Dreher, 2004; Torrance et al., 2006). Another gemini-virus betasatellite-encoded protein, βC1, interacts with PsbP and subverts PsbP-mediated antiviral defense in plants (Gnanasekaran et al., 2019). The beta satellite-mediated impediments at different stages of chloroplast functionality affect the photosynthetic efficiency of *N. benthamiana* (Bhattacharyya et al., 2015). The viral replication complexes targeting to chloroplast require many special chloroplast components. For example, lipid in chloroplast membranes is associate with the localization of Potato mop-top virus (PTMV) encoding protein TGB2 to chloroplast membranes for replication (Cowan et al., 2012). Based on these previous findings, we hypothesized that chloroplastic protein acetylation plays an important role in virus infection.

In this study, large amount of photosynthesis related proteins acetylation were found to be up- or downregulated in *N. benthamiana* leaves infected with CWMV, including several light-harvesting complexes (LHCs) proteins, several PSII subunits and multiple PSI subunits. LHCs are important constituents in light energy transferring of photosystems (Yang et al., 2000). Study in wheat showed that many LHCs located to the chloroplast are associated with photosynthesis, including Lhca1, Lhcb3, Lhcb5, and Lhcb6 (Zhang et al., 2016). PSII is a complete membrane protein complex with more than 20 subunit proteins and a large number of cofactors (Umena et al., 2011). A previous study of *Synechocystis* suggested that PsaD could work with multiple PSI subunits, and suggested that acetylation regulates Fd activity by affecting PsaD interaction with Fd (Mo et al., 2015). Similarly, the acetylation levels of some chloroplast ATP synthases, such as alpha, beta, gamma, and ATPF0B, were different in CWMV-infected plants to those in uninfected plants. The cytochrome complex has previously been shown to affects the ATP and NADPH production by transfer electrons from PSII to PSI (Rowland et al., 2010). Accordingly, we speculate that the acetylated proteins may transfer electrons from PSII to PSI in *N. benthamiana* to participate in photosynthesis.

Protein lysine acetylation (Kac) of histones was first confirmed acetylation event (Phillips, 1963). Here, we found that 19 Kac sites were combined in core histones, including 10 Kac sites on histone 3, which suggests that acetylation plays a role in gene regulation by affecting chromatin structure. Previous reports have shown that a large amount of lysine residues in histone proteins are acetylated, especially histone H3 and histone H4 (Benhamed et al., 2006; Fuchs et al., 2006; Hollender and Liu, 2008; Servet et al., 2010; Tan et al., 2011; Xue et al., 2018). The conservation of histone acetylation among different species highlights the importance of studying the function of histone acetylation in plant biological processes. In addition, a large amount of precise Kac sites of non-histone proteins were demonstrated in this study, which provided valuable information for further studies on the role of protein acetylation in plant-virus interaction.

In conclusion, this is the first extensive dataset of lysine acetylated sites on proteins of CWMV-infected and uninfected *N. benthamiana* plants. A large number of histone lysine residues and many proteins located in the chloroplast are acetylated. Some of these chloroplast proteins are related to photosynthesis, including members of PSI and PSII, as well as different subunits of ATP synthase. This study not only broadens our understanding of lysine acetylation regulates metabolic processes in *N. benthamiana* infected with CWMV but also provides a rich resource for the functional investigation of proteins with Kac sites during virus infection. However, more studies are needed to uncover the role and effects of protein acetylation and to interpret the underlying mechanisms behind protein acetylation in CWMV-infected *N. benthamiana*.

## Data Availability Statement

The datasets generated and analyzed during the current study are available in the ProteomeXchange Consortium via the PRIDE partner repository (https://www.ebi.ac.uk/pride/archive/projects/PXD012537) with the dataset identifier PXD012537.

## Author Contributions

JY and KZ initiated and designed the experiments. BY, KZ, SG, LL, TL, and LC performed the experiments and collected the data. KZ analyzed the data and wrote the manuscript. JY, YC, and JC revised the manuscript. All authors read and approved the final manuscript.

## Conflict of Interest

The authors declare that the research was conducted in the absence of any commercial or financial relationships that could be construed as a potential conflict of interest.

## Footnotes

1 https://www.solgenomics.net/tools/blast/

2 http://www.ebi.ac.uk/GOA/

3 http://www.kegg.jp/kegg/pathway.html

4 http://www.ebi.ac.uk/interpro/

5 https://string-db.org

## Supplementary Material

Supplementary Figure 1 Determination of CWMV infection.
