# Supplementary figures and images for: Comprehensive Proteomic Analysis of Lysine Acetylation in Nicotiana benthamiana After Sensing CWMV Infection
Source: Front Microbiol. 2021 May 17;12:672559. doi: 10.3389/fmicb.2021.672559 (PMC8166574; doi:10.3389/fmicb.2021.672559)

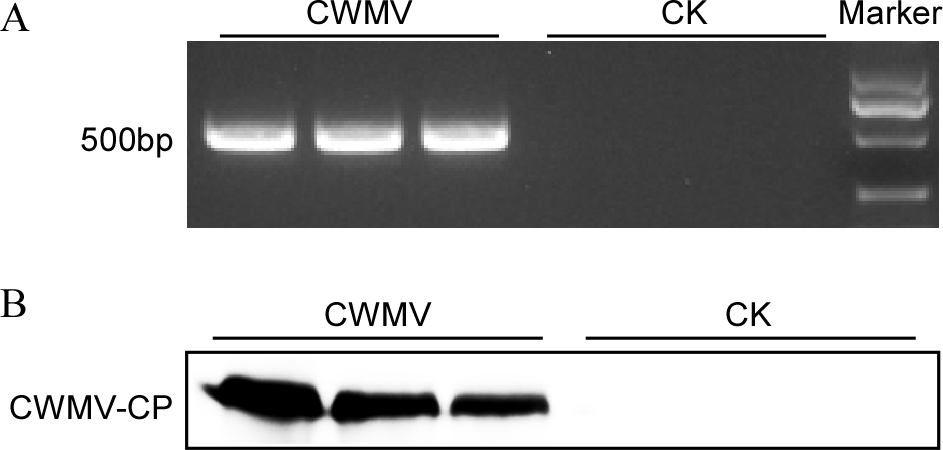

Supplement: Supplementary Figure 1 — Determination of CWMV infection. [file Image_1.TIF]
